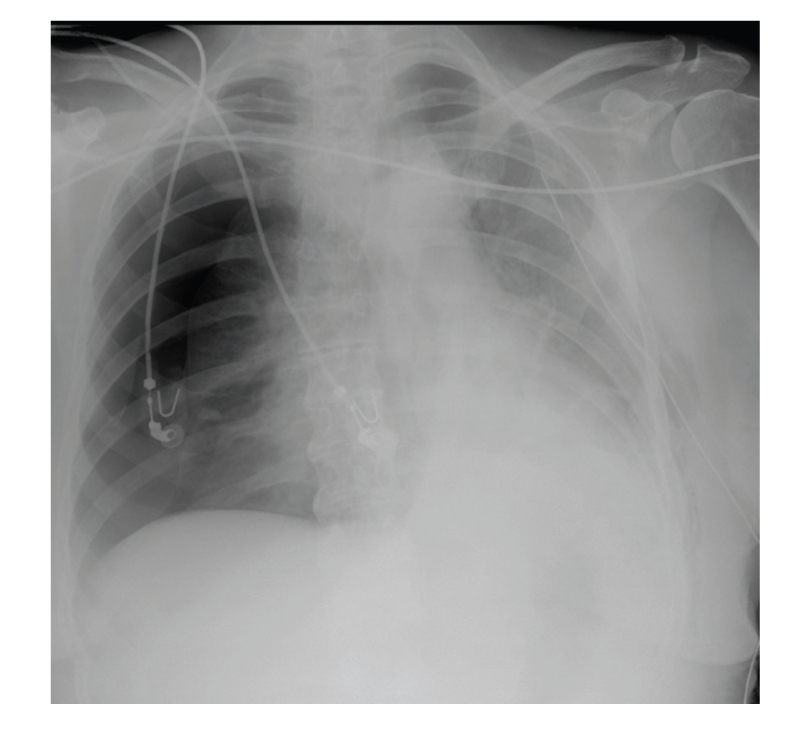# Bilateral Pneumothoraces Following Central Venous Cannulation

**DOI:** 10.1155/2009/745713

**Published:** 2009-11-05

**Authors:** F. Pazos, K. Masterson, C. Inan, J. Robert, B. Walder

**Affiliations:** ^1^Division of Anaesthesiology, University Hospitals of Geneva, CH-1211 Geneva, Switzerland; ^2^Department of Radiology, University Hospitals of Geneva, CH-1211 Geneva, Switzerland; ^3^Division of Thoracic Surgery, University Hospitals of Geneva, CH-1211 Geneva, Switzerland

## Abstract

We report the occurrence of a bilateral pneumothoraces after unilateral central venous catheterization of the right subclavian vein in a 70-year-old patient. The patient had no history of pulmonary or pleural disease and no history of cardiothoracic surgery. Two days earlier, she had a median laparotomy under general and epidural anaesthesia. Prior to the procedure, the patient was hemodynamically stable and her transcutaneous oxygen saturation was 97% in room air. We punctured the right pleural space before cannulation of the right subclavian vein. After the procedure, the patient slowly became hemodynamically instable with respiratory distress. A chest radiograph revealed a complete left-side pneumothorax and a mild right-side pneumothorax. The right-side pneumothorax became under tension after left chest tube insertion. The symptoms finally resolved after insertion of a right chest tube. After a diagnostic work-up, we suspect a congenital “Buffalo chests” explaining bilateral pneumothoraces and a secondary tension pneumothorax.

## 1. Introduction

Pneumothorax is normally a minor mechanical complication of central venous catheterization. It is usually unilateral and occurs on the side of the central line placement. We report a case of bilateral pneumothoraces in a 70-year-old patient after insertion of a central venous catheter in the right subclavian vein. A large work-up could not identify reasons for bilateral pneumothoraces. The most credible hypothesis is the presence of a congenital interpleural communication with the formation of a single pleura described as “Buffalo chests.”

## 2. Case Description

Our IV team was requested to insert a central line for parenteral nutrition in a 70-year-old female patient (weight: 57 kg; height: 160 cm; ASA: 2). Two days earlier, the patient had undergone intestinal and colic resections under general and epidural anaesthesia with a suspected diagnosis of pelvic abscess. No hysterectomy was performed during the intervention. Postoperative diagnosis was an endometrial adenocarcinoma with colic parietal infiltration through to the mucosa.

**Figure 1:**
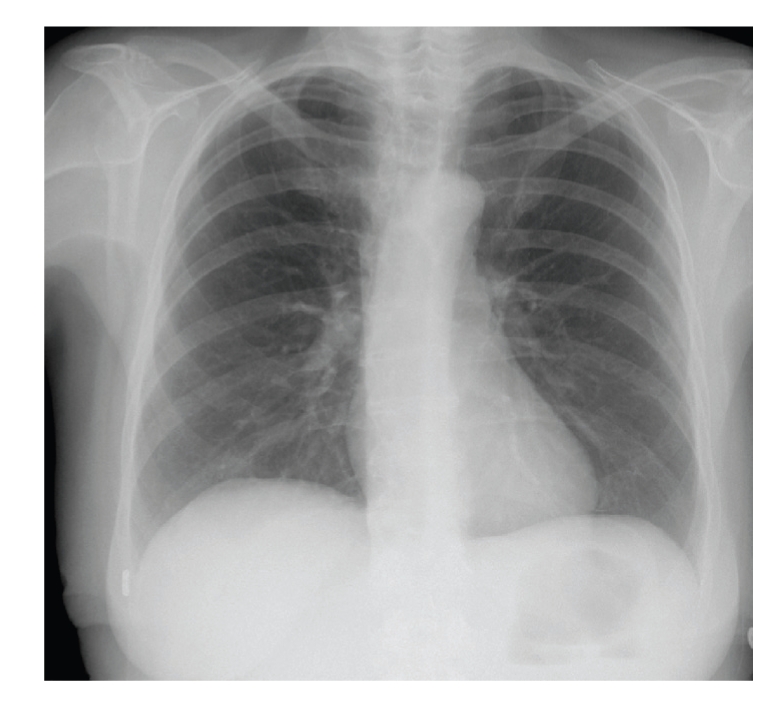


Prior to the procedure, our patient's transcutaneous oxygen saturation was 97% in room air, blood pressure was 135/80 mmHg, and pulse rate was regular at 110/minute. The patient has no history of pulmonary or pleural disease (Figure 1), no history of cardiothoracic surgery or thoracic radiotherapy. No earlier central line placement insertions had been performed.

After placement of noninvasive monitoring a nasal prongs oxygen delivery device was installed administrating 2 L/minute of oxygen. Under maximal aseptic barrier protection a local anaesthesia with 1% lignocaine was placed under the right clavicula and a 18 Gauge introducer needle was introduced mid-clavicularly. The pleural space was punctured twice with the introducer needle after 2.5 cm, confirmed by the presence of air in the syringe. About one minute later, the patient started to complain of pain situated in the right side of the chest that increased at inspiration but without respiratory distress. Her oxygen saturation was at this stage 96%.

Subsequently the site of puncture was moved 2 cm medial compared to the first insertion site and the right subclavian vein was directly punctured at a depth of approximately 3 cm, just under the right-sided sterno-clavicular junction. The entire procedure was completed without any further complications and the patient's oxygen saturation was 94% at the end of the procedure.

**Figure 2:**
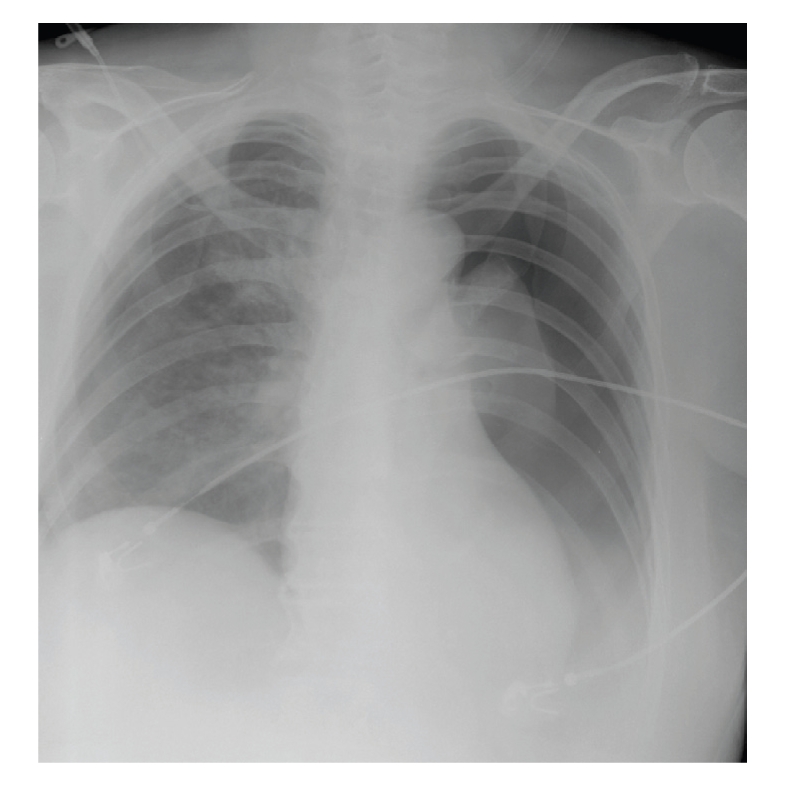


A chest radiograph performed approximately ten minutes after the wound dressing revealed a complete left-side pneumothorax and a mild right-side pneumothorax (Figure 2). At this time, the patient was starting to complain of respiratory distress. Her respiratory rate was 18/minute and her oxygen saturation was 91% with supplemental oxygen of 8 L/minute. Her pulse rate was 140/minute and her blood pressure 144/81 mmHg.

Thoracic surgeons were called and they inserted a chest tube on the left side of the chest. No clinical improvement was noticed and the patient had in between an oxygen saturation of 84% despite a face mask oxygen delivery device with reservoir.

A second chest radiograph was performed and showed a complete right-side tension pneumothorax that was not present on the prior radiograph (Figure 3). The size of the left pneumothorax had decreased after the chest tube insertion. A second chest tube was inserted on the right side and the patient's symptoms resolved and oxygen saturation normalized.

Two days later a computed tomography (CT) of the chest showed persistent bilateral pneumothoraces and leftwards mediastinal shift, the chest tubes were wellplaced, and there were bilateral pleural effusions; furthermore, important bilateral subcutaneous emphysema was present. There was no evidence of emphysema or bulla in the partially expanded nonatelectatic lung parenchyma.

Seven days later, thoracic tubes were removed and one month later, after the first cycle of chemotherapy, the central venous catheter was taken off. Seven days later the patient was discharged without any further complications.

## 3. Discussion

Unilateral pneumothorax is a well-known complication of central venous catheterization and is related to subclavian and jugular accesses. There is some evidence that the risk is similar for both insertion sites [1]. Usually iatrogenic pneumothorax after central venous catheter insertion occurs on the same side as the placement of central venous catheter.

We report a very rare case of simultaneous immediate bilateral pneumothoraces after a right subclavian central venous catheter cannulation.

Three questions in this case need further deepening: What are the potential mechanisms of this bilateral pneumothoraces and what are the potential mechanisms of tension pneumothorax after left-sided drainage in a spontaneous breathing patient? And can this mechanical complication be avoided?

Three potential mechanisms of bilateral pneumothoraces were considered: (1) puncture of a contralateral pulmonary bulla or pleural space, (2) the pre-existence of the contralateral pneumothorax and, (3) the existence of a congenital interpleural communication.

The patient was not known to suffer from lung disease. There were no arguments for a contralateral lung or pleura puncture. We had been working only on the right side and moreover we had never ventured more than approximately 3 cm from the point of skin puncture and we have not crossed the thoracic midline.

Secondly, there were no arguments that a contralateral pneumothorax pre-existed and remained asymptomatic before the occurrence of the second pneumothorax. Two days earlier, the patient has undergone a combined anesthesia including the epidural insertion of a catheter. A few cases of accidental puncture of the lung after thoracic epidural anesthesia have been reported, generally using a thoracic paramedian approach [2, 3]. In our case, the epidural catheter was inserted into the lombar interspace (L1-L2) using a median approach. No ventilatory problems had occurred during the surgery, particularly, there was not high inspiratory pressure (mean peak inspiratory pressure was 15 cm H_2_O). Afterwards, in the postanesthesia care unit, the patient presented no respiratory distress. Her oxygen saturation was 96% with 2 L/minute oxygen some hours after extubation; her pulse pressure was 100/minute and her blood pressure 95/60 mmHg at this time.

Lastly, we can not exclude the existence of a congenital interpleural communication. Normally, the two pleural sacs are separate structures [4] and therefore, a pneumothorax is unilateral, unless a communication exists between each pleural cavity. Communications between each hemithorax have been reported in the literature; they can be congenital [5], iatrogenic essentially following cardiothoracic [6] and even laparoscopic surgery [7, 8], or traumatic in aetiology. These interpleural communications are mainly the consequence of prior median sternotomy [4] with as result the formation of a single pleural space described as “Buffalo chest.” North American buffalos present with a unique pleural sac and a single chest wound would result in bilateral pneumothoraces [5, 6]. Interpleural communications may not be observed on the chest computed tomography if small.

Normally, the diagnosis of “Buffalo chests” can be made if the contralateral lung re-expands after the insertion of a chest tube but the failure of the contralateral lung to re-expand does not necessarily mean that no interpleural communication exists.

How can the right-sided pneumothorax under tension be explained? Check-valve mechanism may occur in a spontaneous ventilating patient, so that a simple pneumothorax may progress in a life-threatening pneumothorax under tension [9–12]. Some cases of spontaneous tension pneumothorax have been described in the literature [9–12]. In spontaneous pneumothoraces, approximately 1-2% of these may be under tension [11]. Unlike to our patient, most of these spontaneous tension pneumothoraces concern healthy young men with no clinical evidence of concurrent respiratory disease and smoking. The cause is thought to be the rupture of subclinical bullae. The peak incidence is between the ages of 20 and 30 years. In some cases, the tension pneumothorax mimics a simple spontaneous pneumothorax and the under tension character of the pneumothorax is revealed only by the radiographies [11].

We have found only one similar case in the literature [13]. A women developed bilateral pneumothoraces after transbronchial biopsies two years after a heart-lung transplantation. The single chest tube drainage failed to re-expand the contralateral hemithorax and resulted in a potentially life-threatening tension pneumothorax. The authors suspected the presence of a small persistent anterior mediastinal communication and that the drainage of the unilateral pneumothrax drew the adjacent scar tissue together producing a valve-like effect, preventing drainage of contralateral hemithorax [13].

Can a pneumothorax during central venous catheterisation insertion be avoided? There is increasing evidence that ultrasound-guided catheterisation decrease pneumothoraces (from 2.4% to 0%) and hematothoraces (from 1.7% to 0%), at least for internal jugular vein cannulation [14]. Visualisation of subclavian vein using ultrasonography is much more tricky, therefore, infraclavicular axillary vein cannulation was proposed. About 93% of infraclavicular axillary veins can be identified with ultrasonography and 96% catheterized successfully [15]; no pneumothorax was observed. This technique could avoid completely the described complication in this case report.

## Figures and Tables

**Figure 3:**